# Trilateral interaction between innervation, leukocyte, and adventitia: a new driver of atherosclerotic plaque formation

**DOI:** 10.1038/s41392-022-01121-9

**Published:** 2022-07-23

**Authors:** Yahya Sohrabi, Holger Reinecke, Oliver Soehnlein

**Affiliations:** 1 Department of Cardiology I—Coronary and Peripheral Vascular Disease, Heart Failure, University Hospital Münster, Westfälische Wilhelms-Universität, Münster, Germany; 2 Institutes of Molecular Genetics of the Czech Academy of Sciences, Prague, Czechia; 3 Institute of Experimental Pathology (ExPat), Center for Molecular Biology of Inflammation (ZMBE), University Hospital Münster, Westfälische Wilhelms-Universität, Münster, Germany; 4 Department of Physiology and Pharmacology (FyFa), Karolinska Institutet, Stockholm, Sweden

**Keywords:** Molecular biology, Cardiology

A recent study by Mohanta et al. describes a new understanding of neuroimmune interactions in atherosclerosis. While atherosclerotic plaques are not innervated, ablating the sympathetic innervation to these regions attenuated atherosclerosis, suggesting a potential novel therapeutic strategy.^1^

Atherosclerosis continues to be the leading cause of death in industrialized countries due to its complications such as acute myocardial infarction or stroke. The disease is the result of a complex process, consisting of sub-endothelial lipid retention, immune cell migration, proteolytic injury, altogether leading to a chronic inflammatory responses in the wall of large arteries and ultimately plaque development.^2^ The inner layer of the blood vessels is not innervated and hence the nervous system connects with the vessel through its most outer layer, the lamina adventitia, hereby controlling important functions including arterial blood pressure. Beyond such homeostatic control of vessel function, increasing evidence demonstrates that activation of the sympathetic nervous system can modulate the degree of arterial inflammation.^3,4^ However, a clear mechanistic foundation of how neuronal signals precipitate arterial disease remains unclear.

Recently, Mohanta et al. have uncovered a complex crosstalk between arteries, nervous system and the immune system in atherosclerosis and termed this neuroimmune cardiovascular interfaces.^1^ Their results demonstrated that—although atherosclerotic plaques themselves are not innervated—axon density in the plaque regions was much higher compared to plaque-free regions. Moreover, axon growth increased with ageing and was significantly more in artery-associated tertiary lymphoid organs (ATLOs)—immune cell aggregates that resemble lymph node-like structures at sites of chronic inflammation—versus non-ATLO regions in *Apoe*^*−/−*^ mice.^1^ Intriguingly, increased plaque and ATLO sizes correlated positively with widespread neuroinflammation in the peripheral nervous system (PNS). In addition, local neuro-vascular inflammation activates distinct areas of the central nervous system (CNS) through an artery–brain circuit (ABC). Interestingly, ABC activation emerged in adulthood and was pronounced during aging in *Apoe*^*−/−*^ mice. Expanded axon networks in the adventitia of aged *Apoe*^*−/−*^ mice provide larger contact spaces for sensory neuron signals thus, larger areas in the CNS are activated. To understand if neuronal signals in turn have a role in atherosclerosis progression, the authors performed chemical denervation of the PNS and surgical celiac ganglion sympathectomy. Astonishingly, the ganglionectomized mice exhibited attenuated atherosclerosis characterized by a reduction in the number, size, and cellularity of ATLOs as well as more stable and smaller plaques. Therefore, the nervous system seems to prevent disease progression by modulating immune cell mobilization and infiltration into the site of inflammation through a hitherto unknown mechanism. Hence, the study by Mohanta et al. contributes mechanistic insight into the tight interplay between innervation and arterial inflammation and may provide a foundation for recently identified drivers of atherosclerosis: acute stress^3,5^ and circadian rhythms.^2,4^

**Fig. 1:**
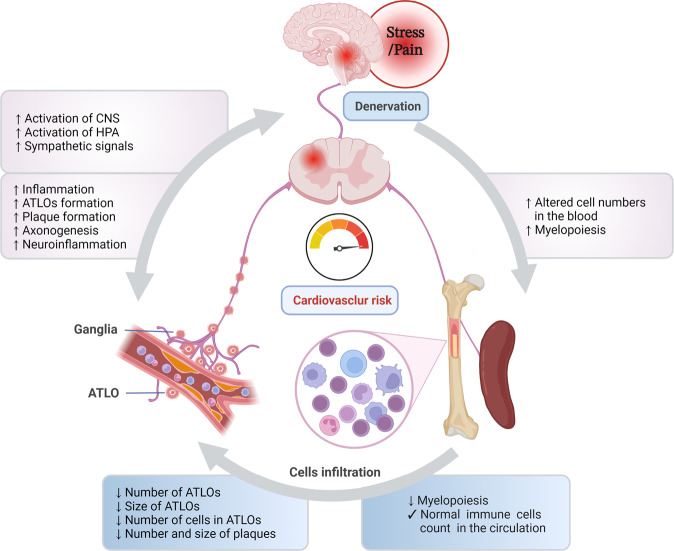
Denervation attenuates atherosclerosis. Stress and pain may trigger atherosclerosis via activating the sympathetic nervous system (SNS) and hypothalamic-pituitary-adrenal (HPA) axis. Mental stress alters immune cell balance in the circulation and promotes myelopoiesis in the bone marrow and spleen. Activation of the SNS promotes artery-associated tertiary lymphoid organ (ATLO) formation and induces axonogenesis around adventitia adjacent to plaque segments. Sympathectomy and denervation reduces atherosclerosis by reducing the number, size, and cellularity of ATLOs as well as plaque formation (indicated by blue color). It also decreases infiltrating cells into the plaques and improves the hematopoietic stem cells profile as well as circulating cell numbers. The figure was created with BioRender.com

In line with the strong epidemiological evidence for the circadian manifestation of cardiovascular complications, leukocyte infiltration of atherosclerotic lesions, as the major driver of atheroprogression, has been shown to be tightly controlled by the circadian clock.^4^ In fact, circadian oscillations of arterial leukocyte recruitment disappear following sympathetic denervation or inhibition of β_2_-adrenergic receptor signaling.^4^ In addition, clinical and epidemiological studies demonstrate that increasing mental stress accelerates the risk of cardiovascular diseases (CVD) and substantially contributes to the onset and progression of atherosclerosis.^3^ Mechanistically, stress aggravates cell influx into the atherosclerotic plaque by modulating endothelial cell activity and infiltration of immune cells into the plaque.^3^ Short-term acute stress is indeed sufficient to activate cell adhesion molecules on endothelial cells and enhance migration of circulating cells to the plaques.^3^ Interestingly, chemical and surgical denervation that altered the norepinephrine signaling reversed endothelial cell activation and stress-induced leukocyte influx into the plaques.^3^ Furthermore, optogenetic and chemogenetic approaches applied in a very recent study revealed that distinct regions in the brain differentially and rapidly control stress-induced fluctuation of immune cells in the circulation, which impairs immune functions and enhances susceptibility to diseases.^5^ In addition to stress, a growing body of evidence confirms the strong reciprocal relationship between nociception and the immune system, which likely influences inflammatory processes through similar mechanisms as stress. Besides sympathetic innervation, pain sensation has recently emerged as an important regulator of the magnitude of inflammation. Likewise, increasing evidence suggests that the intermingled neural circuits controlling both stress and anxiety are more prevalent in patients with CVD and are associated with a worse prognosis of the disease. This attribute may explain why the adults and elderly, who significantly experience more stress, anxiety, and pain as a social group, are more vulnerable to diseases like CVD. Decreasing health, poor sleep quality, high pain scores, vascular aging, and less physical activity during aging enhance stress-derived atherosclerosis by enhancing vascular inflammation, increasing innervation and immune cell infiltration of the atherosclerotic plaques. Altogether, data provided by Mohanta et al. identified a trilateral reciprocal interrelation between the neuronal system, the vasculature, and the immune system that contribute to disease development. Modulation of these interactions may offer new ways to reduce the burden of the ensuing pathology (Fig. 1).

Defining the detailed molecular mechanisms how peripheral inflammation can activate the neuronal system and in turn how the nervous system and risk factors like stress can control immune cell functions at sites of inflammation, will further improve our understanding of atherosclerosis development and treatment. Yet, the study by Mohanta et al. reveals details of the complex anatomical foundation of how neuronal signals may control atheroprogression. Recent advances in biological techniques such as optogenetics and chemogenetics as well as genome editing tools like CRISPR/Cas9 will deepen our understanding of brain-organ communication and will help to identify new therapeutic targets for precision medicine. Psychologic interventions or pharmacological approaches to manage stress and anxiety are associated with decreased risk of CVD. Thus, pharmaceutical drugs that inhibit the noradrenergic signaling like β-blockers, which attenuate the effects of stress hormones are recommended as first-line therapy to improve prognosis after acute coronary syndrome and as secondary prevention of CAD morbidity and mortality in high-risk patients. Mohanta et al. provide an insight into the mechanism of action for noradrenergic signaling in reducing CVD burden. Targeting neurotransmitters such as norepinephrine and multifunctional molecules like Netrin-1 may also be viable new candidates for treating CVD. Furthermore, improving mental health conditions by reducing stress is a new therapeutic frontier to prevent stress-induced CVD. Recently, new direct noninvasive modulation of the vagal nerve using transcutaneous stimulation has emerged as an effective therapeutic approach to a multitude of diseases, including CVD. In addition, abnormal activation of neural circuits regulating mental stress or anxiety are associated with worse disease outcomes. Therefore, therapeutic interventions modulating artery–brain circuits by local denervation could be a potential strategy to attenuate atherosclerosis. Altogether, findings by Mohanta et al. describe a novel mechanism of atherosclerosis development and highlight the neuroimmunological approach as a tool to prevent and treat vascular inflammation and plaque formation.
